# Supplementary material for: Metabolic cross-feeding in imbalanced diets allows gut microbes to improve reproduction and alter host behaviour
Source: Nat Commun. 2020 Aug 25;11:4236. doi: 10.1038/s41467-020-18049-9 (PMC7447780; doi:10.1038/s41467-020-18049-9)
Supplement: Supplementary file 1 — Supplementary Information [file 41467_2020_18049_MOESM1_ESM.pdf]

Supplementary Information

Metabolic cross-feeding in imbalanced diets allows gut microbes to improve reproduction and alter host behaviour

Authors

Sílvia F. Henriques[1], Darshan B. Dhakan[1+], Lúcia Serra[1+], Ana Patrícia Francisco[1], Zita Carvalho-Santos[1], Célia Baltazar[1], Ana Paula Elias[1], Margarida Anjos[1], Tong Zhang[2], Oliver D. K. Maddocks[2], Carlos Ribeiro[*1]

Affiliations

[1] Behavior and Metabolism Laboratory, Champalimaud Research, Champalimaud Centre for the Unknown, Lisbon, 1400-038, Portugal

[2] University of Glasgow Institute of Cancer Sciences, Switchback Road, Glasgow G61 1QH, UK

[+] Authors contributed equally

*Correspondence to:

carlos.ribeiro@neuro.fchampalimaud.org

Supplementary Figures

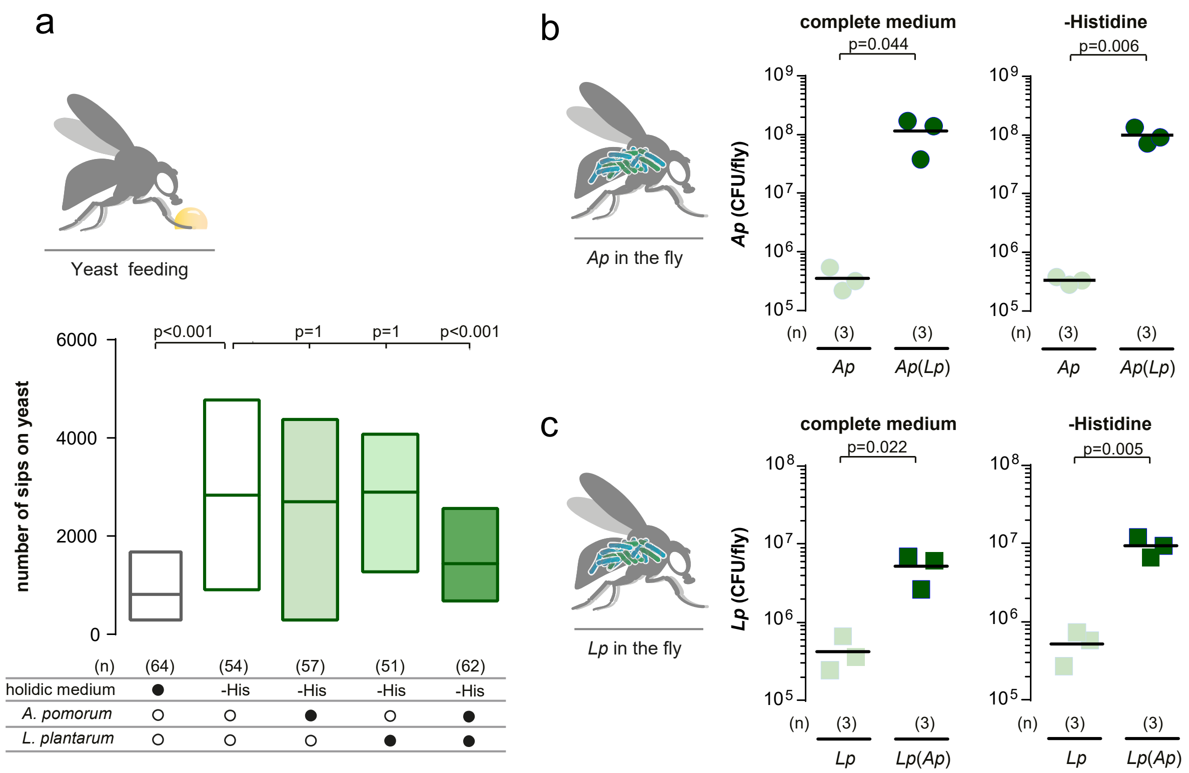

Supplementary Figure 1 – *Ap*/*Lp* biassociation reduces yeast appetite and promotes bacterial growth independently of dietary His content. (**a**) Number of sips on yeast of flies kept in complete holidic medium or medium without His (-His) that were monoassociated (light green) with *Ap* or *Lp*, or biassociated (dark green) with both commensal species. Empty boxes represent germ-free conditions and filled boxes represent bacteria-associated conditions. Boxes represent median with upper and lower quartiles. n represents the number of flies assayed per condition. Significance was tested using a Kruskal–Wallis test with Dunn's multiple comparison test. Filled black circles represent a complete holidic medium or association with specific bacteria. Open black circles represent the absence of specific bacteria. (**b** and **c**) Number of *Ap* (circles, **b**) and *Lp* (squares, **c**) colony-forming units (CFUs) from extracts of monoassociated (light green) and biassociated (dark green) flies kept in complete holidic media, or medium lacking His (-Histidine). *Ap* and *Lp* label data points represent the number of CFUs detected in monoassociated flies and *Ap*(*Lp*) and *Lp*(*Ap*) the measurement of *Ap* or *Lp* in biassociated flies. Each data point represents the number of CFUs detected in independent fly extracts (n), and the black line represents the mean. Significance was tested using an unpaired two-tailed student t-test. Source data are provided as a Source Data file.

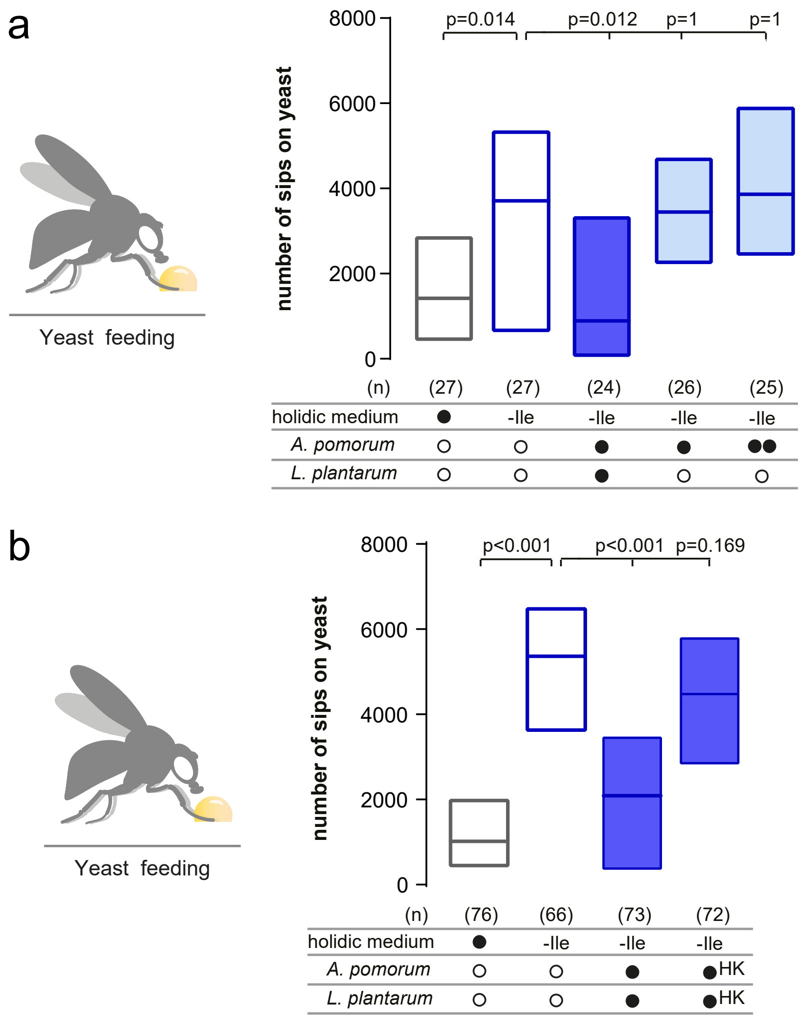

Supplementary Figure 2 – Association with high titres of *Ap* or high amounts of heat-killed bacteria do not reduce the yeast appetite of the fly. (**a** and **b**) Number of sips on yeast of either germ-free flies (empty boxes) kept in complete holidic medium (grey border boxes) or medium without Ile (-Ile, blue border boxes), or flies that were monoassociated (light blue) with *Ap*, or biassociated (dark blue) with both *Ap* and *Lp*. Filled black circles represent a complete holidic medium or association with *Ap* or *Lp*. In (**a**) two filled black circles indicate the association of germ-free flies with a high titre of 10^9 *Ap* cells. In (**b**) HK indicates flies repeatedly treated with Heat-Killed bacteria (1.01 x 10^9 *Ap* and 1.31 x 10^9 *Lp*) throughout the three days of Ile deprivation. (**a** and **b**) Boxes represent median with upper and lower quartiles. n represents the number of flies assayed per condition. Significance was tested using a Kruskal–Wallis test with Dunn's multiple comparison test. Filled black circles represent a complete holidic medium or association with specific bacteria. Open black circles represent the absence of specific bacteria. Source data are provided as a Source Data file.

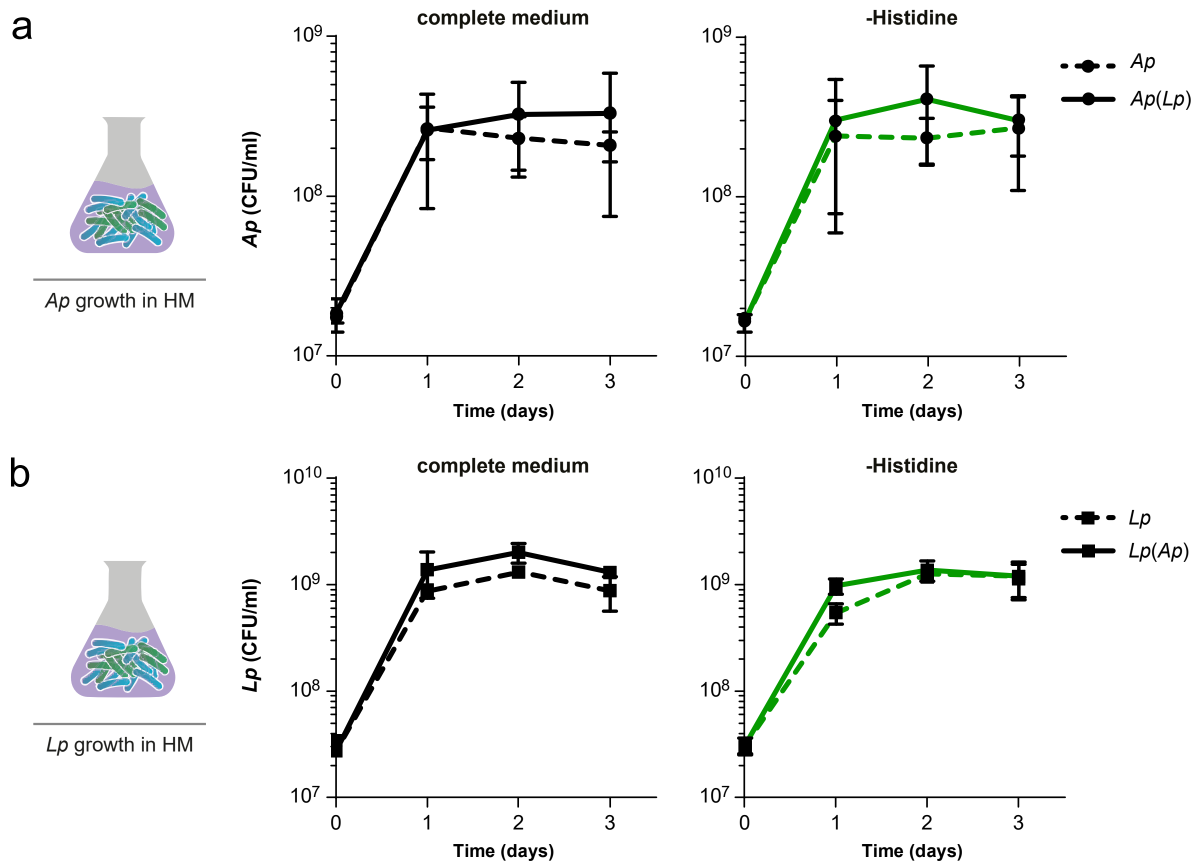

Supplementary Figure 3 – *In vitro* growth measurements show that removing His from the diet does not affect *Ap* or *Lp* growth. *In vitro* growth curves of *Ap* (**a**) and *Lp* (**b**) in complete liquid holidic medium (black line) or liquid medium without His (green line) plotted as CFUs at different time intervals. Data points connected by dashed lines represent the number of CFUs detected in cultures of individual species (*Ap* or *Lp*) and the data points connected by continuous lines, the number of *Ap* (labelled as *Ap*(*Lp*)) or *Lp* (labelled as *Lp*(*Ap*)) CFUs in co-culture conditions. The number of CFUs per ml of culture was determined by cultivating liquid culture samples in selective media at the indicated time points. For each condition, three independent bacterial cultures were sampled at 0 h, 24 h, 48 h, and 72 h, except for the *Lp* single culture grown in media lacking His for which four cultures were sampled. Each data point represents the mean CFUs from independent bacterial cultures and the error bars the standard deviation (s.d.). Source data are provided as Source data file.

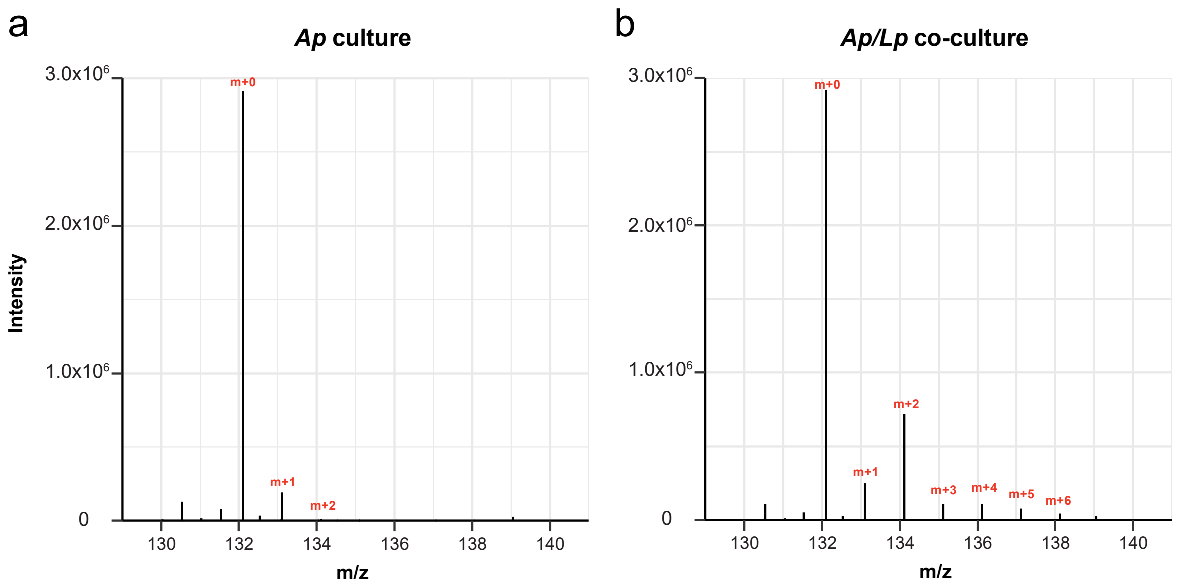

Supplementary Figure 4 – Raw mass spectra of Ile from *Ap* single culture and *Ap*/*Lp* co-culture. Raw unprocessed mass spectra of Isoleucine in positive ion mode is shown for **(a)** an *Ap* single culture and **(b)** an *Ap*/*Lp* co-culture that were cultivated for 24 h in holidic medium lacking Ile and containing ^{13}C labelled glucose. The mass spectra represent peaks at the retention time of 730 s (12.1 mins) for each individual isotopologues (^{13}C labelled) of Ile. The x-axis represents mass/charge ratio (m/z), whereas the y-axis represents the raw intensity values (without corrections) of the identified isotopologues of Ile.

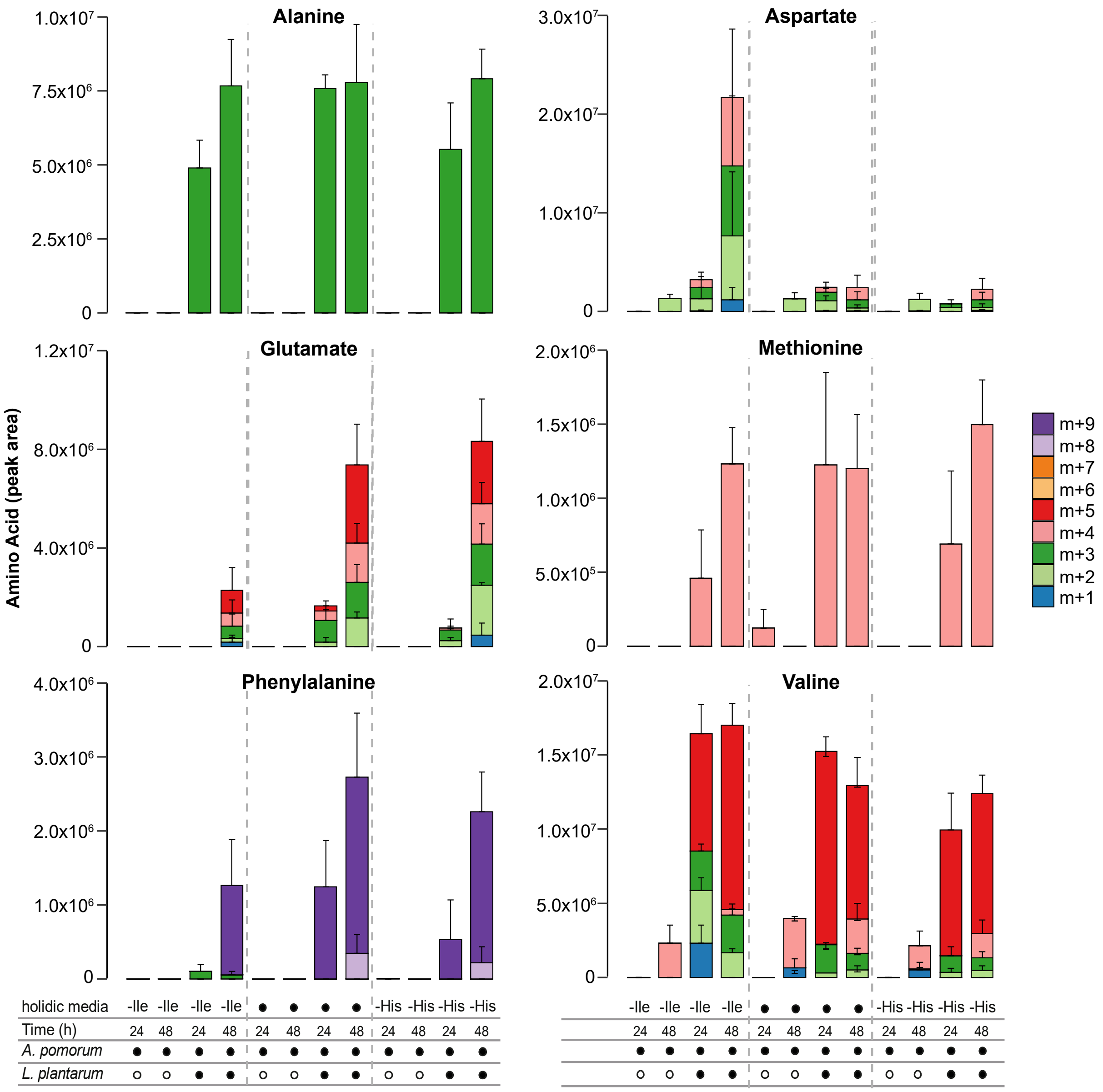

Supplementary Figure 5 – Synthesis of AAs is increased in the presence of *Lp*. The stacked bars represent the relative amount of isotopologues of each indicated AA measured in the supernatant of bacterial cultures grown in either media lacking Ile (-Ile), or in complete liquid holidic medium (filled black circles), or media lacking His (-His), containing uniformly ^{13}C labelled glucose. Heavy-labelled isotopologues were measured using LC-MS in samples collected after 24 h and 48 h of bacterial growth and displayed as metabolite peak area. The number of heavy carbons incorporated per molecule is indicated as m+n, where m = molecular mass of the metabolite and n = the number of ^{13}C. Filled black circles represent the presence and open black circles the absence of specific bacteria. For each condition, three independent bacterial cultures were sampled. The plots represent the mean values of the amount of the detected isotopologues per condition and the error bars represent the s.e.m.. Source data are provided as a Source Data file.

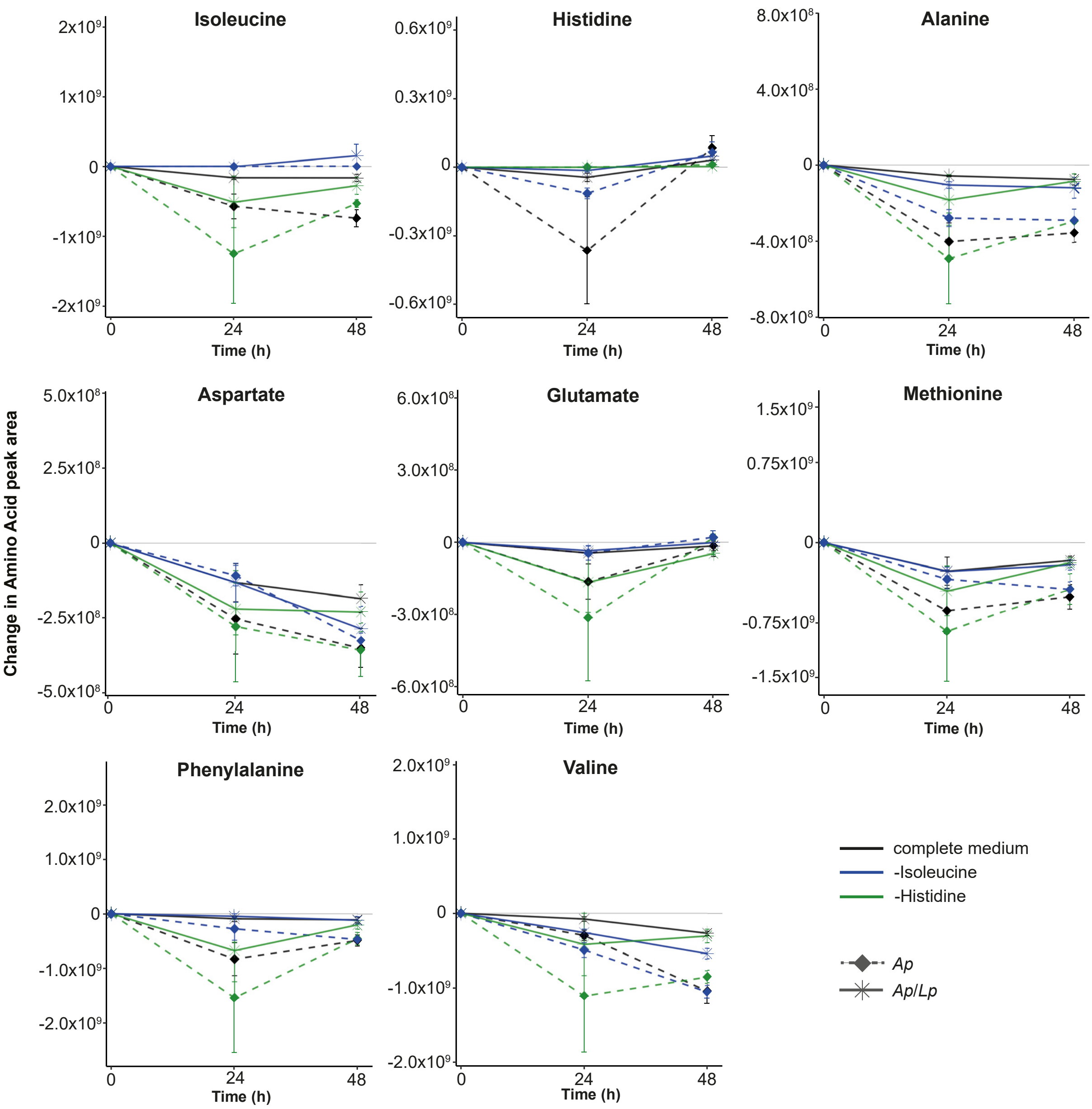

Supplementary Figure 6 – Commensal bacteria utilize amino acids in the HM. Each data point represents the change in the amount of different unlabelled (m+0) AAs in the bacterial culture compared to the amount found in control media conditions (without bacterial growth). Single cultures of *Ap* (diamonds connected with dashed line) and co-cultures of *Ap*/*Lp* (stars connected with a continuous line) were grown in complete holidic medium (black lines) and medium lacking either Ile (blue lines) or His (green lines) containing uniformly ^{13}C labelled Glucose. Non-labelled AAs (m+0) were measured using LC-MS in samples collected after 0 h, 24 h, and 48 h of bacterial growth and displayed as metabolite peak area. Data are from the same dataset as Fig. 3 and Supplementary Figure 5. For each condition, three independent bacterial cultures were sampled. Each data point represents the mean change in the amount of the detected AAs per condition and the error bars represent the s.e.m.. Source data are provided as a Source Data file.

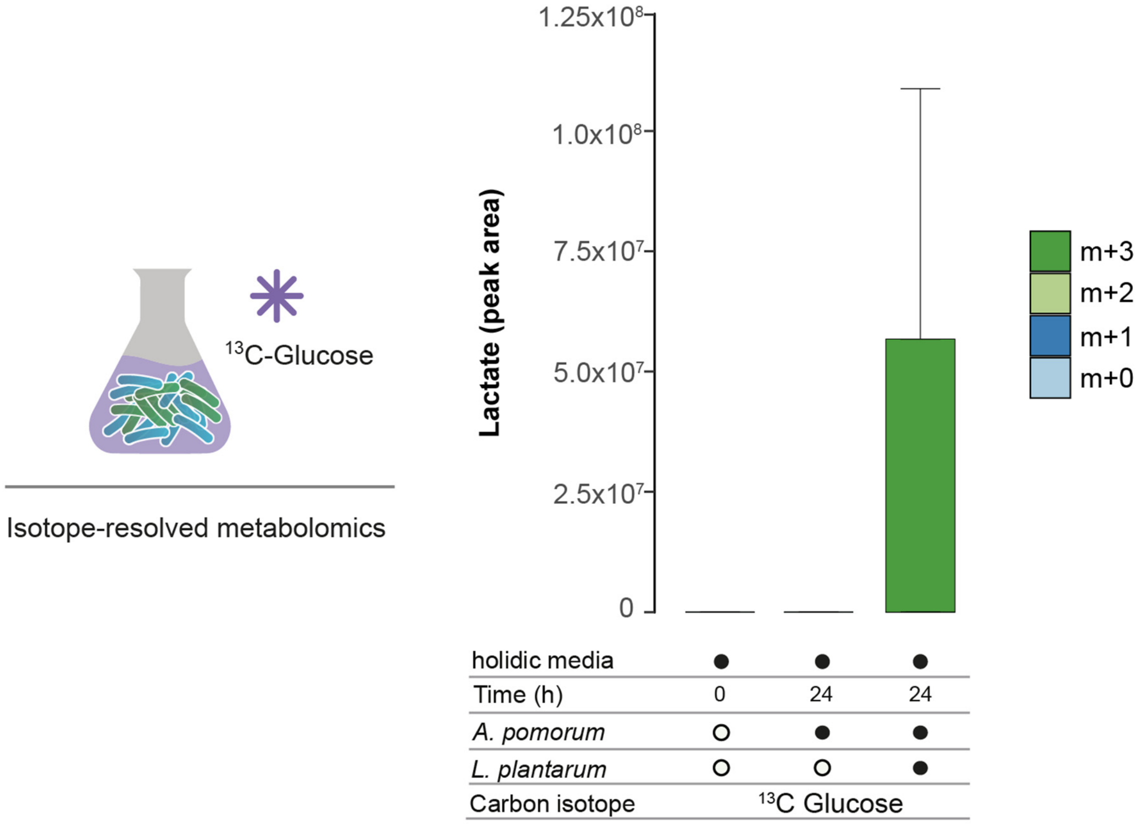

Supplementary Figure 7 – Lactate is produced in *Lp* cultures. Bars represent the relative amount of heavy lactate measured in the supernatant of bacterial cultures grown in complete liquid holidic medium containing uniformly ^{13}C labelled glucose (^{13}C-glucose). Heavy labelled lactate was measured using LC-MS in samples collected after 0 h or 24 h of bacterial growth and displayed as metabolite peak area. The number of heavy carbons incorporated per molecule is indicated as m+n, where m = molecular mass of the metabolite and n = the number of ^{13}C. Filled black circles represent the presence of specific bacteria in the culture. Open black circles represent the absence of specific bacteria. For each condition, three independent bacterial cultures were sampled. The plots represent the mean amount of the detected lactate isotopologues per condition and the error bars represent the s.e.m.. Source data are provided as a Source Data file.

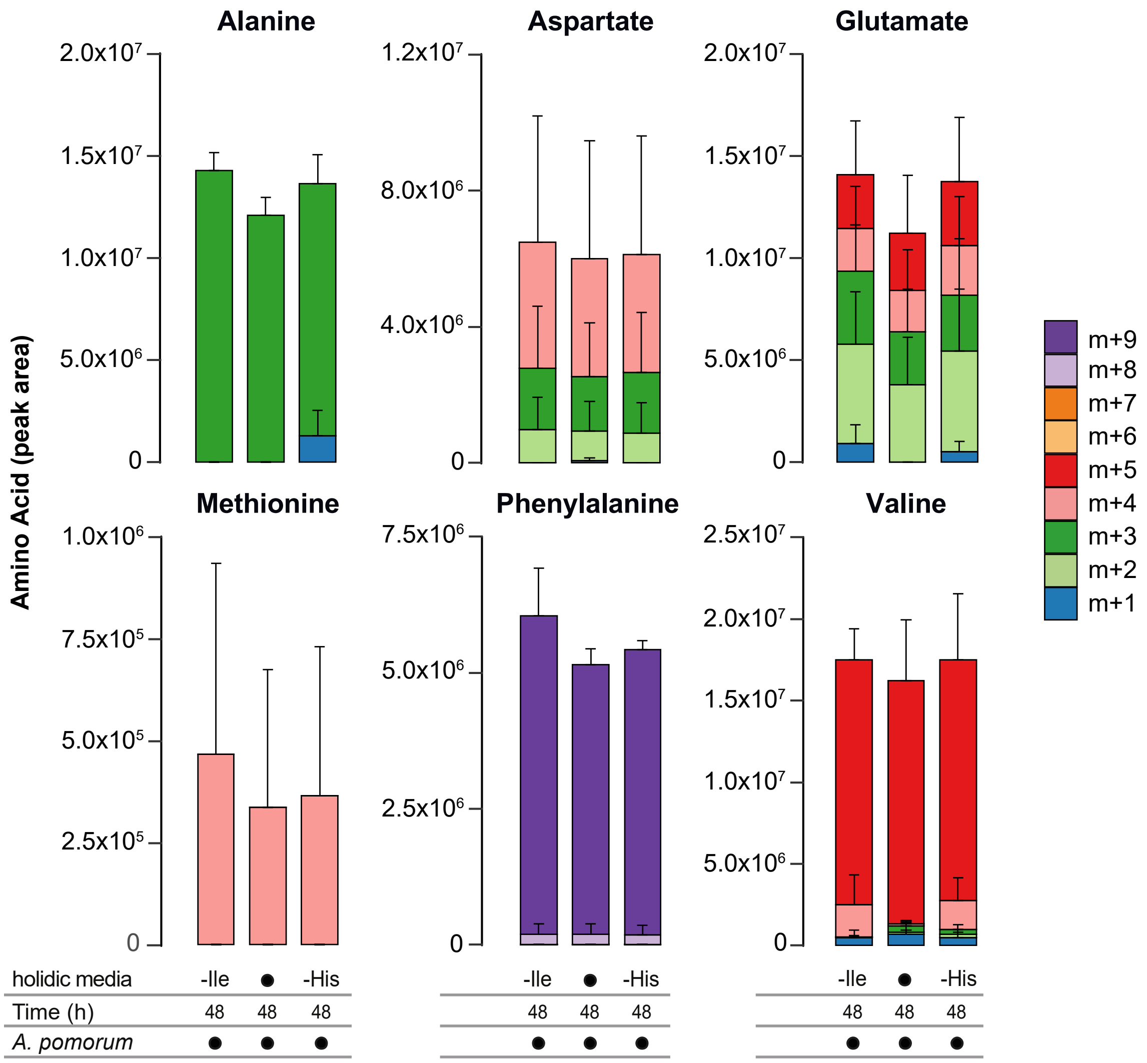

Supplementary Figure 8 – Secreted AAs are synthesized from lactate. The stacked bars represent the relative amount of isotopologues of each indicated AA measured in the supernatant of bacterial cultures grown in complete liquid holidic medium (filled black circles) or in media lacking Ile (-Ile) or His (-His) containing uniformly ^{13}C labelled lactate (^{13}C-lactate). Heavy labelled isotopologues were measured using LC-MS in samples collected after 48 h of bacterial growth and displayed as metabolite peak area. The number of heavy carbons incorporated per molecule is indicated as m+n, where m = molecular mass of the metabolite and n = the number of ^{13}C. Filled black circles represent the presence of specific bacteria in the culture. For each condition, three independent bacterial cultures were sampled. The plots represent the mean amounts of the detected isotopologues per condition and the error bars represent the s.e.m.. Source data are provided as a Source Data file.

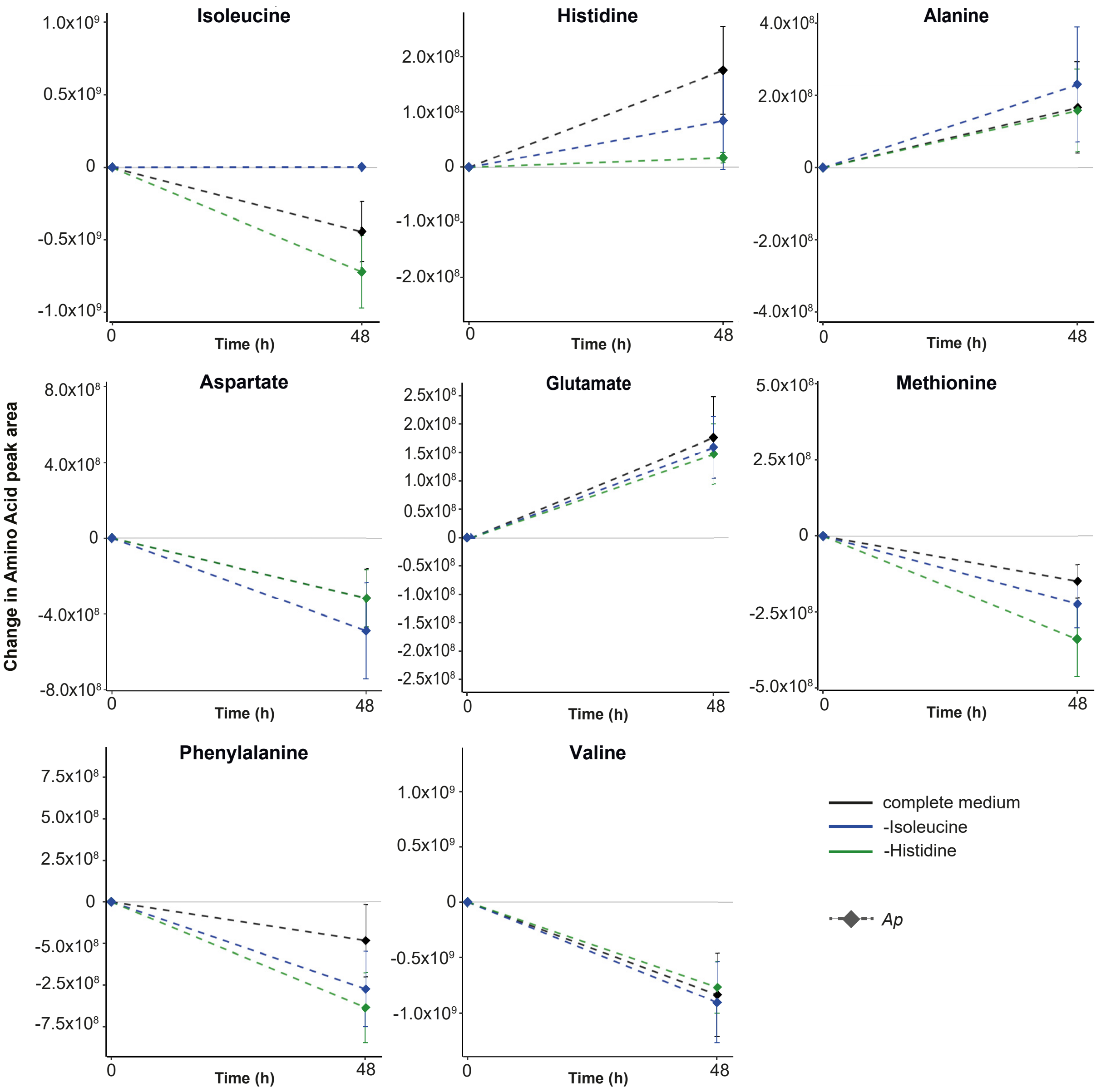

Supplementary Figure 9 – Commensal bacteria utilize amino acids in the HM (data using ^{13}C-lactate). Each data point represents the change in the amount of different unlabelled (m+0) AAs in the bacterial culture compared to the amount found in control media conditions (without bacterial growth). Single cultures of *Ap* (diamonds connected with dashed line) were grown in complete holidic medium (black lines) and medium lacking either Ile (blue lines) or His (green lines) containing uniformly ^{13}C labelled Lactate. Non-labelled AAs (m+0) were measured using LC-MS in samples collected after 0 h and 48 h of bacterial growth and displayed as metabolite peak area. Data are from the same dataset as Fig. 5a and Supplementary Figure 8. Each data point represents the mean change in the amount of the detected AAs per condition and the error bars represent the s.e.m.. Source data are provided as a Source Data file.

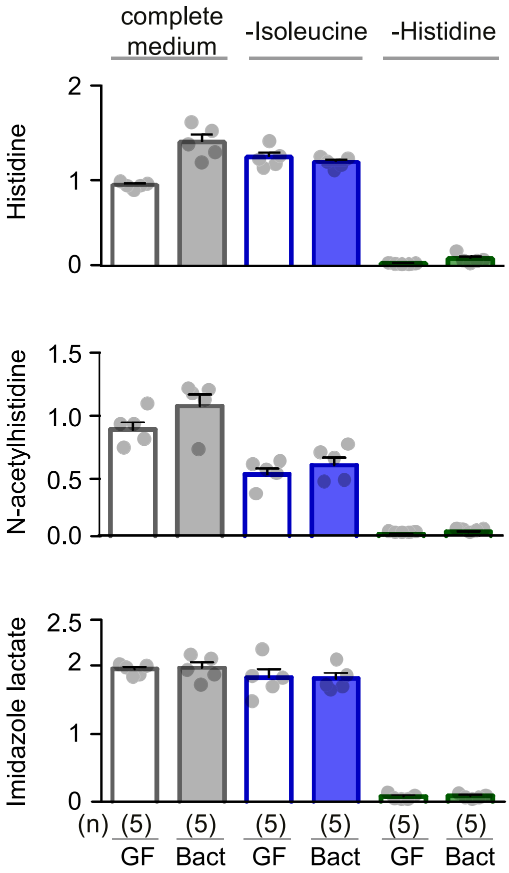

Supplementary Figure 10 – His and His degradation products are not increased in heads of flies inoculated with bacteria. Bars represent the mean relative abundance of His and His degradation metabolites, as measured in metabolomics experiments from heads of germ-free flies (GF) and flies inoculated with commensal bacteria (Bact) and maintained in either complete holidic medium (grey boxes), medium lacking Ile (blue boxes), or medium lacking His (green boxes). Grey circles represent measurements of metabolites in independent extracts of heads of flies (n), the columns represent the mean and the error bars the s.d.. Source data are provided as a Source Data file.

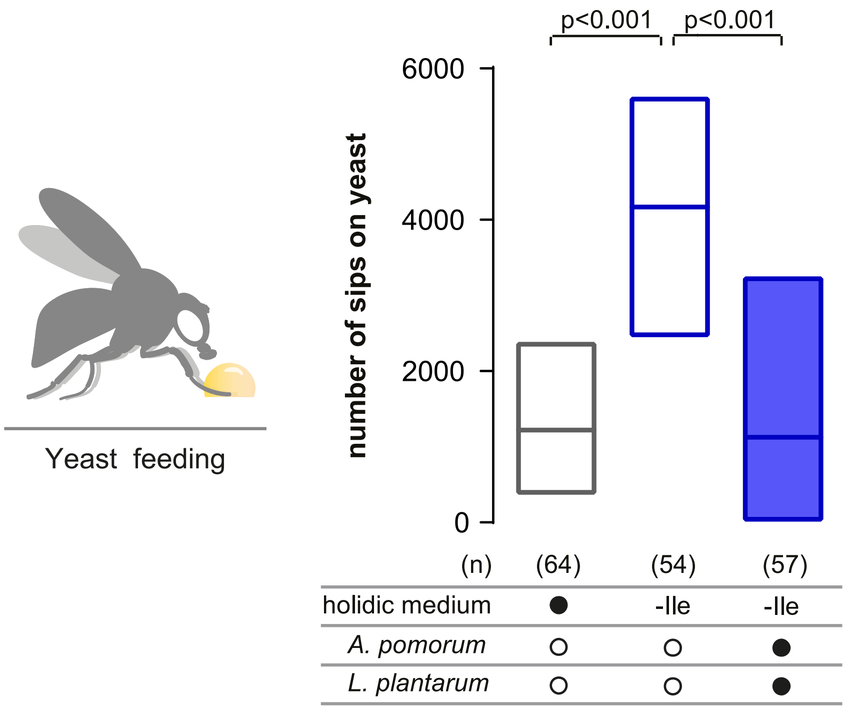

Supplementary Figure 11 – Glucose and sucrose affect fly's yeast preferences to the same extent. Number of sips on yeast of germ-free (empty boxes) flies or flies biassociated (blue boxes) with *Ap* and *Lp* that were kept in complete holidic medium or medium without Ile (-Ile) where an equimolar concentration of the monosaccharide glucose (100 mM) was used as a carbon source in the holidic medium (see Methods section), instead of the disaccharide sucrose (50 mM). Boxes represent median with upper and lower quartiles. n represents the number of flies assayed per condition. Significance was tested using a Kruskal–Wallis test with Dunn's multiple comparison test. Filled black circles represent a complete holidic medium or association with *Ap*/*Lp*. Open black circles represent the absence of bacteria. Source data are provided as a Source Data file.

Supplementary Tables

Supplementary Table 1. Enzymes predicted to mediate the biosynthesis of Ile from lactate in the *Acetobacter pomorum* strain DM001. Information used to infer the pathway depicted in Figure 5b.

Reaction Step	Enzyme Name	Reaction	Gene Name	KEGG reference ID
**1**	D-lactate dehydrogenase (cytochrome) [EC:1.1.2.4]	Lactate + NAD^+ => Pyruvate + NADH	WP_006115901.1	apk:APA386 B_1053
**1**	D-lactate dehydrogenase [EC:1.1.1.28]	Lactate + NAD^+ => Pyruvate + NADH	WP_035350829.1	apk:APA386 B_910
**2**	pyruvate dehydrogenase E1 component subunit beta [EC:1.2.4.1]	Pyruvate + CoA + NAD+ => Acetyl CoA + NADH + CO_2	WP_035354176.1	apk:APA386 B_2084
**2**	pyruvate dehydrogenase E1 component subunit beta [EC:1.2.4.1]	Pyruvate + CoA + NAD+ => Acetyl CoA + NADH + CO_2	WP_035351377.1	apk:APA386 B_2737
**2**	pyruvate dehydrogenase E1 component subunit alpha [EC:1.2.4.1]	Pyruvate + CoA + NAD+ => Acetyl CoA + NADH + CO_2	WP_006115287.1	apk:APA386 B_2738
**2**	pyruvate dehydrogenase E1 component subunit alpha [EC:1.2.4.1]	Pyruvate + CoA + NAD+ => Acetyl CoA + NADH + CO_2	WP_035354178.1	apw:APA42 C_05940
**2**	pyruvate dehydrogenase E2 component (dihydrolipoamide acetyltransferase) [EC:2.3.1.12]	Pyruvate + CoA + NAD+ => Acetyl CoA + NADH + CO_2	WP_006115285.1	apk:APA386 B_2736
**2**	pyruvate dehydrogenase E2 component (dihydrolipoamide acetyltransferase) [EC:2.3.1.12]	Pyruvate + CoA + NAD+ => Acetyl CoA + NADH + CO_2	WP_006117117.1	apk:APA386 B_2085
**3**	citrate synthase [EC:2.3.3.1]	Acetyl CoA + Oxaloacetate + H_2O => Citrate + CoA + H+	WP_035351757.1	apk:APA386 B_2584
**4**	aspartate aminotransferase [EC:2.6.1.1]	Oxaloacetate + Glutamate => Aspartate + 2-oxoglutarate	WP_006117475.1	apk:APA386 B_861
**4**	aspartate aminotransferase [EC:2.6.1.1]	Oxaloacetate + Glutamate => Aspartate + 2-oxoglutarate	WP_035351899.1	apk:APA386 B_862
**5**	aspartate kinase [EC:2.7.2.4]	Aspartate + ATP => Aspartyl 4-phosphate + ADP	WP_035351732.1	apw:APA42 C_11270
**6**	homoserine dehydrogenase [EC:1.1.1.3]	Aspartate semialdehyde + NAD(P)H + H^+ => Homoserine + $NAD(P)^+$	WP_006115936.1	apk:APA386 B_992
**7**	homoserine kinase Type II [EC:2.7.1.39]	Homoserine + ATP => O-phospho Homoserine + ADP + H+	WP_006117496.1	apw:APA42 C_19760

**8**	threonine synthase [EC:4.2.3.1]	O-phospho Homoserine + H_2O => Threonine + Phosphate	WP_006116529.1	apk:APA386B_602
**9**	threonine dehydratase [EC:4.3.1.19]	Threonine => 2-aminobut-2-enoate => 2-oxobutanoate	WP_035351562.1	apw:APA42C_09850
**10**	acetolactate synthase I/II/III large subunit [EC:2.2.1.6]	pyruvate + 2-oxobutanoate => 2-aceto,2-hydroxybutanaote + CO_2	WP_035351917.1	apk:APA386B_836
**10**	acetolactate synthase I/II/III large subunit [EC:2.2.1.6]	pyruvate + 2-oxobutanoate => 2-aceto,2-hydroxybutanaote + CO_2	WP_035352550.1	apw:APA42C_03810
**11**	ketol-acid reductoisomerase [EC:1.1.1.86]	2-aceto,2-hydroxybutanoate + NADPH => 2,3-dihydroxy,3-methylpentanoate + $NADP^+$	WP_035351970.1	apk:APA386B_834
**12**	dihydroxy-acid dehydratase [EC:4.2.1.9]	2,3-dihydroxy,3-methylpentanoate => 3-methy,2-oxopentanoate	WP_035352258.1	apk:APA386B_1291
**13**	branched-chain amino acid aminotransferase [EC:2.6.1.42]	3-methyl, 2-oxopentanoate + Glutamate => Isoleucine + 2-oxoglutarate	WP_006117469.1	apk:APA386B_855
**13**	branched-chain amino acid aminotransferase [EC:2.6.1.42]	3-methyl, 2-oxopentanoate + Glutamate => Isoleucine + 2-oxoglutarate	WP_035353342.1	apk:APA386B_1001
