# Supplementary material for: Metabolic cross-feeding in imbalanced diets allows gut microbes to improve reproduction and alter host behaviour
Source: Nat Commun. 2020 Aug 25;11:4236. doi: 10.1038/s41467-020-18049-9 (PMC7447780; doi:10.1038/s41467-020-18049-9)
Supplement: Supplementary file 3 — Description of Additional Supplementary Files [file 41467_2020_18049_MOESM3_ESM.docx]

**Description of Supplementary Files**

**File Name: Supplementary Data 1**

**Description:** List of the values of the areas under curve (AUC) for each analyzed metabolite isotopologue after the pre-processing of the raw mass spectrometry data. The following information is provided: sample name (“Sample”); identified metabolite (“Metabolite”); description of cultivated bacteria (“Bacteria”; “Blank”, ”Ap” or “Ap/Lp”); used labelled substrate (“Labelled substrate”; “13C_Glucose”, ”13C_Lactate”); dietary content of the medium used in the culture (“Media”); incubation time of the culture (“Time”; 0h, 24h, 48h); grouping of samples (“Group”); the analysed metabolite isotopologue (”isotopologue”); raw area under the curve of the peaks after xcms pre-processing (“AUC”); area under the curve corrected for natural isotopologue abundance using Isocor (“Corrected AUC”); area under the curve normalized using the OD600 value for differences in bacterial growth (“Normalized AUC”); fraction of the isotopologue within all isotopologues of the analysed metabolite (“Isotopologue fraction”); mean percent enrichment of 13C in the analysed metabolite (“Mean Percent Enrichment”); error after correction for natural isotopologue abundance (“residuum”).”
