# Supplementary material for: Metabolic cross-feeding in imbalanced diets allows gut microbes to improve reproduction and alter host behaviour
Source: Nat Commun. 2020 Aug 25;11:4236. doi: 10.1038/s41467-020-18049-9 (PMC7447780; doi:10.1038/s41467-020-18049-9)
Supplement: Supplementary file 5 — Reporting Summary [file 41467_2020_18049_MOESM5_ESM.pdf]

nature research

Corresponding author(s): Carlos Ribeiro

Last updated by author(s): Jul 13, 2020

Reporting Summary

Nature Research wishes to improve the reproducibility of the work that we publish. This form provides structure for consistency and transparency in reporting. For further information on Nature Research policies, see Authors & Referees and the Editorial Policy Checklist .

Statistics

For all statistical analyses, confirm that the following items are present in the figure legend, table legend, main text, or Methods section.

n/a	Confirmed	
☐	☒	The exact sample size (*n*) for each experimental group/condition, given as a discrete number and unit of measurement
☐	☒	A statement on whether measurements were taken from distinct samples or whether the same sample was measured repeatedly
☐	☒	The statistical test(s) used AND whether they are one- or two-sided<br>*Only common tests should be described solely by name; describe more complex techniques in the Methods section.*
☐	☒	A description of all covariates tested
☐	☒	A description of any assumptions or corrections, such as tests of normality and adjustment for multiple comparisons
☐	☒	A full description of the statistical parameters including central tendency (e.g. means) or other basic estimates (e.g. regression coefficient) AND variation (e.g. standard deviation) or associated estimates of uncertainty (e.g. confidence intervals)
☐	☒	For null hypothesis testing, the test statistic (e.g. *F*, *t*, *r*) with confidence intervals, effect sizes, degrees of freedom and *P* value noted<br>*Give P values as exact values whenever suitable.*
☒	☐	For Bayesian analysis, information on the choice of priors and Markov chain Monte Carlo settings
☒	☐	For hierarchical and complex designs, identification of the appropriate level for tests and full reporting of outcomes
☒	☐	Estimates of effect sizes (e.g. Cohen's *d*, Pearson's *r*), indicating how they were calculated

Our web collection on statistics for biologists contains articles on many of the points above.

Software and code

Policy information about availability of computer code

Data collection	We used generic software and published code for behavioral data acquisition based on Bonsai framework (versions 2.1.4; 2.3; 2.3.1) (https://bonsai-rx.org/) as described in Itskov P et al., Nature communications, 2014 and flypad.pt webpage
Data analysis	Generic software (Graphpad Prism 6) was used for data analysis and statistical analysis. For behavioral analysis, published Matlab codes were used as described in Itskov P et al., Nature communications, 2014 and made available at flypad.pt. The LC-MS data was converted and analyzed by LCquan (thermo Scientific) and processed using xcms v.4 in R for metabolite identification and quantification.

For manuscripts utilizing custom algorithms or software that are central to the research but not yet described in published literature, software must be made available to editors/reviewers. We strongly encourage code deposition in a community repository (e.g. GitHub). See the Nature Research guidelines for submitting code & software for further information.

Data

Policy information about availability of data

All manuscripts must include a data availability statement. This statement should provide the following information, where applicable:

- Accession codes, unique identifiers, or web links for publicly available datasets
- A list of figures that have associated raw data
- A description of any restrictions on data availability

The authors declare that the main data supporting the findings of this study are available within the article and its Supplementary Information files. KEGG (v_2014) (https://www.genome.jp/kegg/kegg1.html) and BioCyc (https://biocyc.org/) databases were used to annotate the enzymes involved in Isoleucine biosynthesis from the genome of Acetobacter pomorum strain DM001 in order to infer the biosynthetic pathway for Isoleucine in this organism. These links are now also available in the "Data availability" section. Source data used to test for statistical significance in figures is also provided as Source Data.

Field-specific reporting

Please select the one below that is the best fit for your research. If you are not sure, read the appropriate sections before making your selection.

[x] Life sciences   [ ] Behavioural & social sciences   [ ] Ecological, evolutionary & environmental sciences

For a reference copy of the document with all sections, see nature.com/documents/nr-reporting-summary-flat.pdf

Life sciences study design

All studies must disclose on these points even when the disclosure is negative.

Sample size	We used standard values according to the general use in the field and determined by our expertise over the last years (Itskov et al, 2014 Nature communications; Leitão-Gonçalves et al, 2017 PLoS Biology; Steck et al 2018 eLife, Scopelliti et al. 2018 Cell Metabolism).
Data exclusions	No data was excluded from the analysis.
Replication	All key behavioral data shown were successfully replicated independently at least twice.
Randomization	Animals were allocated randomly to different dietary conditions and bacterial treatments.
Blinding	Egg laying assays were performed blind to dietary condition and bacterial treatment. In all other experiments experimenters were not blinded to dietary or genotype condition. The analysis of these experiments, including the behavioral assays, the bacterial growth curves, colony counts and the metabolite measurements are done by computer algorithms and as such should not be subjected to investigator bias.

Reporting for specific materials, systems and methods

We require information from authors about some types of materials, experimental systems and methods used in many studies. Here, indicate whether each material, system or method listed is relevant to your study. If you are not sure if a list item applies to your research, read the appropriate section before selecting a response.

Materials & experimental systems

n/a	Involved in the study	
[x]	[ ]	Antibodies
[x]	[ ]	Eukaryotic cell lines
[x]	[ ]	Palaeontology
[ ]	[x]	Animals and other organisms
[x]	[ ]	Human research participants
[x]	[ ]	Clinical data

Methods

n/a	Involved in the study	
[x]	[ ]	ChIP-seq
[x]	[ ]	Flow cytometry
[x]	[ ]	MRI-based neuroimaging

Animals and other organisms

Policy information about studies involving animals; ARRIVE guidelines recommended for reporting animal research

Laboratory animals	The Drosophila melanogaster w1118 strain was used in this study. As decribed in the "Methods" section, 6-10-year-old mated female flies were used in all experiments conducted in this study.
Wild animals	No field collected animals or samples were used in this study.
Field-collected samples	No field collected animals or samples were used in this study.
Ethics oversight	The described research activities involved experimentation on fruit flies (Drosophila melanogaster) as a model organism, thereby eliminating the use of protected animals (3Rs). The European Council Directive 86/609/EEC updated and replaced by Directive 2010/63/EU of the European Parliament and of the Council of 22 September 2010, in full effect since 1 January 2013, does not apply to insects. Insects are not live non-human vertebrate animals, nor live cephalopods. Nonetheless, the number of flies used were the minimum necessary to achieve the objectives and the best practices were employed by the researcher and staff working at the Fly Facility. Flies were kept according to applicable European and Institutional guidelines, and persons handling strains and stocks received special training in the appropriate procedures.

Note that full information on the approval of the study protocol must also be provided in the manuscript.
